# Optimization of Communication Tasks in an Energy-Efficient Swarm and the Spatial Distribution of Robots

**DOI:** 10.3390/s26154742

**Published:** 2026-07-26

**Authors:** Amir Ijaz, Hashem Haghbayan, Ethiopia Nigussie, Juha Plosila

**Affiliations:** 1 Department of Computing, University of Turku, FI-20014 Turku, Finland; 2 Faculty of Technology, University of Turku, FI-20014 Turku, Finland

**Keywords:** internet of things (IoT), sustainability, energy optimization, power management, communications, swarm robotics, spatial distribution

## Abstract

Energy-efficient coordination of robotic swarms requires effective integration of task scheduling, motion planning, and communication management, particularly in resource-constrained environments where computation and wireless communication compete for limited energy resources. Existing multi-robot approaches typically address these concerns in separate stages: task-allocation methods (e.g., market- and auction-based schemes) price assignments by distance, and computation-offloading methods decide execution placement after a route has been fixed. This paper’s specific contribution is to fold the execution-placement decision (local computation versus offloading to a peer) into the edge-relaxation step of an A* path search, using a composite cost whose communication term is derived from the instantaneous neighborhood of each node; routing and compute placement are therefore co-optimized within a single search rather than in decoupled stages. The framework is evaluated in simulation with a swarm of 25 robots against two decoupled baselines: a path-only planner that ignores workload and communication costs, and a workload-only scheduler that ignores travel and communication costs. Across 20 randomized trials, the proposed heuristic reduces total swarm energy consumption by approximately 22% relative to the path-only baseline and 9% relative to the workload-only baseline, shortens average task completion time by roughly 20%, and lowers the load imbalance factor from 6.7 (path-only) and 3.2 (workload-only) to 1.9. We report these gains for the tested configurations and delimit their scope: the search retains the asymptotic complexity of standard A*, but path optimality does not extend to the compute-placement decisions, which are locally greedy, and all results are obtained in simulation rather than on hardware.

## 1. Introduction

**Figure 1:**
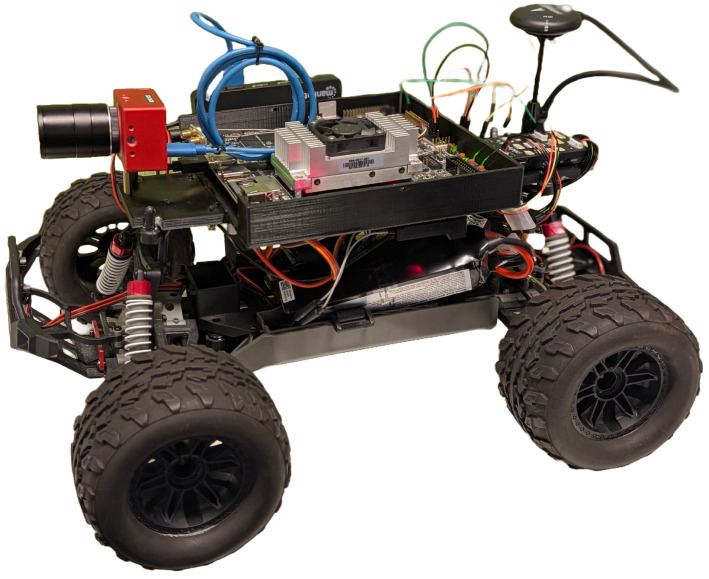
Prototype of rover.

Swarm robotics draws inspiration from the collective behavior of social insects, deploying large numbers of simple agents to accomplish complex tasks through decentralized interaction [1]. A persistent challenge in such systems is the scheduling of communication-intensive tasks [2]: assignments must be distributed across the swarm over time to maximize mission efficiency [3] while respecting hard resource constraints such as battery capacity and communication range [4]. Spatial distribution is equally critical: robot placement governs communication latency, collision risk, and the feasibility of tasks that require close physical proximity [5]. Figure 1 shows the rover prototype that defines the target hardware class for this work. We emphasize that all experiments reported in this paper are simulation studies: the physical prototype was not used to generate the results, and hardware validation on this platform is planned as future work.

Conventional task scheduling approaches [6] typically assume centralized decision-making and largely ignore the spatial dynamics of swarm operation [7]. Such centralized methods are prone to scalability bottlenecks and single-point failures [8]. Conversely, purely spatial algorithms [9], such as coverage control or formation planning, generally abstract away the temporal dimension of task allocation [10]. This paper addresses the intersection of these two domains by proposing a heuristic framework that jointly optimizes task assignment and path planning, explicitly accounting for the costs of spatial distribution and inter-robot communication.

### Research Gap

Reviewing this literature identifies not merely a topic that is understudied, but specific mechanisms by which existing approaches fail to meet the requirements of energy-constrained, communication-aware swarms. Three failure modes recur across the methods surveyed in Section 2:

1. Centralized and market-based schedulers price tasks by distance alone. Auction and utility-based frameworks [11,12] adapt well to unpredictable task arrivals, but their bidding functions typically reduce communication and computation cost to a simple distance metric [13], so a robot that is spatially close but computationally saturated, or communication-starved, is scored the same as one that is not.
2. Spatial and formation-control methods abstract away scheduling. Coverage control and formation-maintenance techniques [14,15] optimize geometric configuration but assume homogeneous, already-scheduled workloads [16], leaving the question of which robot executes which task, and where, outside their scope.
3. Joint formulations exist but decide placement after routing, not during it. Centralized mixed-integer programs [17] and heuristic multi-objective methods, including genetic algorithms [18], ant colony optimization [19], and multi-objective A* variants [20], can in principle balance several objectives at once, but in practice treat communication delay or compute placement as a secondary objective evaluated after a route is fixed [21], or omit an explicit, calibratable communication-energy model altogether [22]. Even where communication topology has recently been folded into scheduling decisions [23], the joint optimization of travel paths together with that topology remains largely unaddressed [24].

None of these method families make the local-versus-offload decision inside the path-search step itself, using a communication cost that is instantaneously sensitive to swarm topology. This is the specific gap addressed in this paper.

Our contributions are threefold:

1. We formally define the joint scheduling and spatial allocation problem for homogeneous, energy-constrained robot swarms operating under communication constraints.
2. We introduce a composite cost metric that integrates travel distance, computational load, and communication overhead (including energy cost), and embed this metric within an A*-based heuristic search.
3. We demonstrate through simulation against two decoupled baselines that the proposed approach reduces total swarm energy consumption by approximately 22% (versus path-only planning) and 9% (versus workload-only scheduling), shortens average task completion time by roughly 20%, and substantially improves workload fairness.

Although the A* algorithm and multi-criteria cost functions are individually well established, the novelty of this work lies in how they are combined to close the gap identified above. First, the execution-mode decision (local computation versus offloading to a peer) is made inside the edge-relaxation step of the search itself, so that each candidate path is priced with the best achievable compute placement along it; existing multi-objective A* variants price edges with fixed attributes and decide task placement in a separate stage. Second, the communication term of the composite cost is derived from the instantaneous neighborhood of each node, which makes the resulting plans sensitive to swarm topology rather than only to geometry. Third, the planner operates in a closed loop with task reassignment, so that placement decisions are revised as the topology changes rather than fixed at dispatch time. To the best of our knowledge, this in-search coupling of routing and computation placement under an explicit communication-energy model has not been examined in the swarm robotics literature.

The remainder of this paper is structured as follows. Section 2 surveys related work. Section 3 presents the proposed framework: its overall architecture, the formal problem statement and assumptions, the environment and task model, the composite cost formulation, the augmented A* algorithm and its computational complexity, and the experimental setup used to evaluate it. Section 4 reports the resulting experiments, covering both the effect of spatial distribution on computational cost and the comparison against baseline strategies. Section 5 discusses these results and their implications, including the limitations of the present study. Section 6 concludes with directions for future work.

## 2. Related Work

Task scheduling in multi-robot systems has received substantial research attention [25]. Early methods relied on centralized planners to assign tasks in a manner that minimizes energy consumption [26]. While these approaches can achieve near-optimal allocations, they introduce scalability bottlenecks and single-point failure risks in large swarms [27]. To overcome these limitations, distributed alternatives, notably market-based and auction-based frameworks, were proposed [11]. In such schemes, robots bid for tasks according to utility functions that may encode travel cost, battery longevity, or computational demand [12]. Although these methods improve reliability and adapt to unpredictable task arrivals, they typically represent spatial factors only through simple distance metrics [13]. Recent centralized architectures continue this trajectory: Mokhtari et al. [28] propose a meta-heuristic, queue-based scheduler for energy-harvesting robot swarms that reduces task completion time relative to energy-unaware baselines, but, like its predecessors, the scheduler dispatches tasks to a fixed robot before routing is determined, rather than jointly pricing route and execution location.

Parallel research has addressed the spatial organization and formation control of robot swarms [29]. Formation control methods maintain geometric configurations [14] to support coordinated monitoring or transportation tasks [15]. However, these spatial strategies generally assume homogeneous workloads and overlook the costs of fine-grained scheduling and communication [16]. In the UAV domain, Alqudsi and Makaraci [30] integrate task allocation with collision-free trajectory generation for drone swarms, demonstrating that joint treatment of assignment and motion improves mission completion relative to sequential allocate-then-plan pipelines; their formulation, however, does not model computation placement or an explicit communication-energy cost, focusing instead on geometric feasibility.

More recently, efforts have begun to integrate task allocation with spatial planning [31]. Centralized mixed-integer programming formulations can jointly optimize assignment and routing [17], but their computational complexity precludes real-time use. Heuristic approaches, including genetic algorithms [18], ant colony optimization [19], and multi-objective A* variants [20], offer more tractable solutions but often treat communication delay or workload distribution as secondary objectives [21]. In swarm-specific settings, bio-inspired mechanisms such as response threshold models exploit local interactions to balance workload, yet typically omit explicit communication costs and dynamic path replanning [22]. Recent work has begun to incorporate communication topology into scheduling decisions [23], but joint optimization of travel paths remains largely unaddressed [24]. Ferreira et al. [32] extend distributed scheduling to cross-schedule task dependencies in heterogeneous multi-robot teams, improving robustness to inter-task precedence constraints, but their allocator operates independently of the robots’ physical routing.

Closest in spirit to the present work are recent studies that couple communication cost directly with task or computation decisions. Palieri et al. [33] propose an SDN-inspired orchestrator that offloads perception workloads (e.g., lidar or visual odometry) to more capable peers in a heterogeneous robot team, using a centrally computed, globally aware schedule; this validates the practical benefit of peer-to-peer computation offloading in robot teams, but the offloading decision is made by a central orchestrator rather than embedded in each robot’s own path search, and travel routing is not part of the optimization. Yasser et al. [34] design decentralized swarm communication algorithms that jointly target task allocation and power consumption, showing that communication-aware clustering reduces energy use relative to unclustered baselines; their algorithms operate over a fixed task graph, however, and do not integrate a path-planning search, so the interaction between motion cost and communication cost that is central to the present formulation is not modeled.

To make the positioning concrete, Table 1 contrasts the main method families against the three axes most relevant to this work: whether travel routing is optimized, whether computation placement (offloading) is decided, and whether an explicit communication-cost model couples the two. Classical multi-robot task allocation (MRTA) taxonomies [29] and consensus-based auction methods of the CBBA family [11,12] excel at distributed assignment but treat the winning robot’s route and its computation placement as downstream concerns. Mobile edge computing (MEC) offloading work, including the vehicular and cloud-resource frameworks the reviewer highlights [35,36,37], develops sophisticated, often learning-based, decisions about where computation executes and how resources are allocated, but assumes the requesting agent’s mobility/route is given rather than jointly planned; the secure trajectory-prediction-plus-offloading scheme of Wu et al. [35] couples predicted motion with offloading, but for single vehicles served by fixed roadside units rather than a peer swarm co-optimizing its own routes. Communication-aware routing and coding methods [38] optimize how data traverses a network under channel constraints, but do not decide task-to-robot assignment or physical navigation. The present work is distinguished by occupying the intersection of all three axes within a single search step.

In contrast, the present work explicitly models a composite cost metric that combines travel distance, local computation effort, and inter-robot communication overhead, embedding this metric within an A*-based heuristic so that routing and compute-placement decisions are made jointly, inside the same search step, rather than in separate stages. Once a task is assigned to a robot, that robot’s routing and local-versus-offload execution decisions (Algorithm 1) are made using only locally observable neighborhood information (Section 3.7); the task-assignment step itself (Algorithm 2) ranks candidate robot–task pairs swarm-wide and therefore requires centralized visibility as currently implemented, a scope boundary we state precisely, rather than obscure, in Section 3.7. We report the per-query computational complexity of the local routing step in Section 3.6 and note that wall-clock latency on embedded hardware has not yet been measured (Section 5.2), so “real-time” is used here only in the algorithmic-complexity sense and not as a validated timing guarantee.

**Algorithm 1** Augmented A* with dynamic compute offload decision.
**Require:** Graph G=(V,E), edge costs $d_{ij}$, start *s*, goal *g***Require:** Workload map w(·), neighbors N(v), comm. cost $c_{vv^{\prime }}$, weights α,β,γ>0**Ensure:** Path *P* and decision map computeDecision(v)∈{local,offload}
     1:**function** OffloadCost(*v*)     2:     **if** N(v)=⌀ **then return** +∞     3:     **elsereturn** $\gamma \min_{v^{\prime }\in N\left(v\right)}c_{vv^{\prime }}$     4:     **end if**     5:
**end function**
     6:**function** Heuristic(u,g) **return** $\alpha \;{\mathrm{dist}}_{euclid}\left(u,g\right)$     7:
**end function**
     8:**for all** u∈V **do**     9:     g[u]←+∞, parent[u]←nil   10:
**end for**
   11:g[s]←0, f[s]← Heuristic(s,g)   12:Open←{(s,f[s])}, Closed←⌀   13:computeDecision[·]← empty   14:**while** Open≠⌀ **do**   15:     u←PopMin(Open)   16:     **if** u=g **then break**   17:     **end if**   18:     Closed←Closed∪{u}   19:     **for all** v∈N(u) **do**   20:         **if** v∈Closed **then continue**   21:         **end if**   22:         $C_{t}\leftarrow \alpha d_{uv}$, $C_{l}\leftarrow \beta w_{v}$, $C_{o}\leftarrow$ OffloadCost(*v*)   23:         $C_{c}\leftarrow \min\left(C_{l},C_{o}\right)$   24:         $g^{\prime }\leftarrow g\left[u\right]+C_{t}+C_{c}$   25:         **if** $g^{\prime }<g\left[v\right]$ **then**   26:              $g\left[v\right]\leftarrow g^{\prime }$, parent[v]←u   27:              f[v]←g[v]+Heuristic(v,g)   28:              Update/Insert (v,f[v]) in Open   29:              $\mathrm{computeDecision}\left[v\right]\leftarrow \left\{\begin{array}{cc} \mathrm{local}, & C_{l}\leq C_{o}\\ \mathrm{offload}, & \mathrm{otherwise} \end{array}\right.$   30:         **end if**   31:     **end for**   32:
**end while**
   33:**if** parent[g]=nil **then return failure**   34:
**end if**
   35:

P←[g]

   36:**while** P[0]≠s **do**   37:     Prepend parent[P[0]] to *P*   38:
**end while**
   39:**return** (P,computeDecision)


**Algorithm 2** Multi-robot task allocation with communication-aware planning (outer loop).
**Require:** Robots R with positions $p_{r}$, capacities $Q_{r}$; unassigned tasks T; weights α,β,γ
**Note:** Steps 2–3 require swarm-wide visibility of R and T (centralized coordinator, or an equivalent full broadcast); see Section 3.7
**Ensure:** Assignments, collision-free paths $\left\{P_{r}\right\}$, and compute decisions for all executed tasks
     1:**while** T≠⌀ **do**                                                                        ▹ one scheduling iteration per pass     2:      Update neighborhoods N(·) and queue states from current beacons                 ▹ Section 3.7     3:      $C\leftarrow \{\left(r,j,\;C_{total}\left(r,j\right)\right):r\in R,\;j\in T\}$                                   ▹ *centralized*: requires global R,T     4:      Sort C ascending by $C_{total}$                                            ▹ *centralized* ranking step, Equation (17)     5:      A←⌀                                                                                                 ▹ assignments this iteration     6:      **for all** (r,j,·)∈C in sorted order **do**     7:          **if** *j* unassigned **and** $|\left\{j^{\prime }:\left(r,j^{\prime }\right)\in A\right\}|<Q_{r}$ **then**     8:             A←A∪{(r,j)}                                                               ▹ greedy commit; enforces (6), (7)     9:          **end if**   10:      **end for**   11:      Res←⌀                                                                                          ▹ space-time reservation table   12:      **for all** robots *r* with assignments in *A*, in priority order **do**   13:          $(P_{r},{\mathrm{dec}}_{r})\leftarrow$ Algorithm 1 on *G* with cells in Res blocked   14:          Res←Res∪ space-time cells of $P_{r}$                                                     ▹ enforces (11) and (12)   15:      **end for**   16:      Execute one round: robots follow $\left\{P_{r}\right\}$, performing local execution or offload per ${\mathrm{dec}}_{r}$   17:      Remove completed tasks from T; update positions, queues, energy accounts   18:
**end while**
   19:**return** all assignments, paths, and compute decisions


## 3. Proposed Framework

This section describes the proposed communication-aware planning framework. It is organized to separate architecture from formulation from procedure: Section 3.1 gives the high-level design; Section 3.2 states the problem formally and lists the assumptions under which it is solved; Section 3.3 defines the environment and task representation; Section 3.4 derives the composite cost, including the physical communication and energy models, as a single unified formulation; Section 3.5 presents the augmented A* algorithm that consumes this cost; Section 3.6 analyzes its computational complexity; and Section 3.8 describes the experimental setup used to evaluate the framework in Section 4. Each concept below is introduced once; its performance consequences are reported and discussed only in Section 4 and Section 5.

### 3.1. Overall Framework Architecture

**Figure 2:**
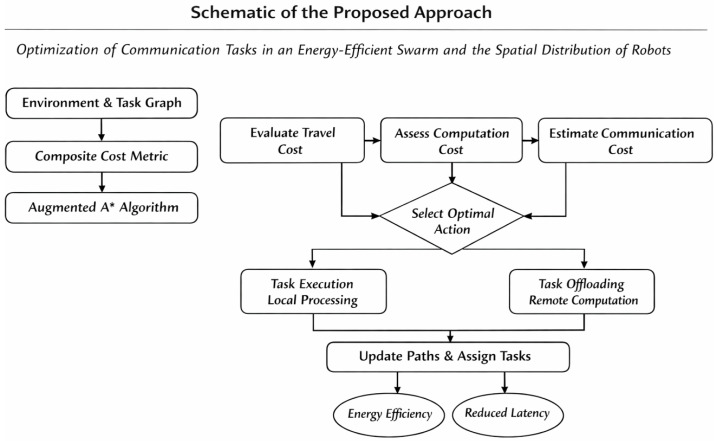
Overview of the proposed co-optimization approach.

The framework’s central design choice is to treat travel, computation, and communication as a single joint cost, rather than as three problems solved in sequence (Figure 2). Three components implement this choice. First, the environment and outstanding tasks are represented as a graph, described in Section 3.3. Second, a composite cost metric (Section 3.4) prices every candidate edge traversal jointly with the cheaper of two execution modes at the destination node: computing the workload locally, or offloading it to a communication-reachable neighbor. Third, an augmented A* search (Section 3.5) consumes this composite cost during edge relaxation, so that the local-versus-offload decision is made as part of path planning rather than afterward. After each execution step, task assignments and trajectories are refined in a closed loop, so that early decisions do not lock the swarm into a configuration that later becomes suboptimal as the topology changes.

### 3.2. Problem Formulation and Assumptions

#### 3.2.1. Sets and Parameters

Let $R=\{r_{1},\ldots ,r_{N}\}$ be the set of homogeneous robots and T={1,…,M} the set of tasks, each task *j* located at position $p_{j}$ with input data size $D_{j}$ (bits) and workload $w_{j}$. Each robot *r* has an initial position $p_{r}^{0}$, a designated return/end position $p_{r}^{\mathrm{end}}$, an initial energy budget $B_{r}$ (J), and a processing capacity $Q_{r}$ (maximum number of concurrently queued tasks). Two robots (or a robot and a task location) are in communication range when their Euclidean separation does not exceed the communication radius $r_{c}$; the time-varying neighbor set of node *v* is $N\left(v\right)=\{v^{\prime }:∥p_{v}-p_{v^{\prime }}∥\leq r_{c}\}$.

#### 3.2.2. Decision Variables


(1) $$\begin{array}{cccc} x_{r,j} & \in \{0,1\}, & \; & \mathrm{task}\;j\;\mathrm{is}\;\mathrm{assigned}\;\mathrm{to}\;\mathrm{robot}\;r; \end{array}$$

(2) $$\begin{array}{cccc} y_{r,i,j} & \in \{0,1\}, & \; & \mathrm{robot}\;r\;\mathrm{travels}\;\mathrm{directly}\;\mathrm{from}\;\mathrm{node}\;i\;\mathrm{to}\;\mathrm{node}\;j\;(\mathrm{path}/\mathrm{routing}\;\mathrm{variable}); \end{array}$$

(3) $$\begin{array}{cccc} z_{j} & \in \{0,1\}, & \; & \mathrm{task}\;j\;\mathrm{is}\;\mathrm{offloaded}\;(z_{j}=1)\;\mathrm{rather}\;\mathrm{than}\;\mathrm{executed}\;\mathrm{locally}\;(z_{j}=0); \end{array}$$

(4) $$\begin{array}{cccc} o_{j,r^{\prime }} & \in \{0,1\}, & \; & \mathrm{offloaded}\;\mathrm{task}\;j\;\mathrm{is}\;\mathrm{executed}\;\mathrm{by}\;\mathrm{neighbor}\;r^{\prime }. \end{array}$$

#### 3.2.3. Objective

Minimize the total swarm cost, aggregated over travel, computation, and communication:

(5) $$\min_{x,y,z,o}\;\sum_{r\in R}\sum_{\left(i,j\right)}\alpha \;d_{ij}\;y_{r,i,j}\;+\;\sum_{j\in T}\left(1-z_{j}\right)\;\beta \;w_{j}\;+\;\sum_{j\in T}z_{j}\;\gamma \;\;\min_{r^{\prime }\in N\left(j\right)}c_{j,r^{\prime }}.$$

#### 3.2.4. Constraints

(6) $$\begin{array}{cccccc} \sum_{r\in R}x_{r,j} & =1 & \; & \forall j\in T & \; & (\mathrm{each}\;\mathrm{task}\;\mathrm{assigned}\;\mathrm{once}) \end{array}$$

(7) $$\begin{array}{cccccc} \sum_{j\in T}x_{r,j} & \leq Q_{r} & \; & \forall r\in R & \; & (\mathrm{task}\text{-}\mathrm{capacity}\;\mathrm{limit}) \end{array}$$

(8) $$\begin{array}{cccccc} \sum_{i}y_{r,i,j} & =\sum_{k}y_{r,j,k} & \; & \forall r,\forall j & \; & (\mathrm{route}\;\mathrm{continuity}/\mathrm{flow}\;\mathrm{conservation}) \end{array}$$

(9) $$\begin{array}{cccccc} o_{j,r^{\prime }} & \leq z_{j}\;⊮\left[\;r^{\prime }\in N\left(j\right)\;\right] & \; & \forall j,\forall r^{\prime } & \; & (\mathrm{offload}\;\mathrm{only}\;\mathrm{within}\;\mathrm{range}) \end{array}$$

(10) $$\begin{array}{cccccc} E_{r}^{\mathrm{move}}+E_{r}^{\mathrm{cpu}}+E_{r}^{\mathrm{comm}} & \leq B_{r} & \; & \forall r\in R & \; & (\mathrm{per}\text{-}\mathrm{robot}\;\mathrm{energy}/\mathrm{battery}\;\mathrm{budget}) \end{array}$$

(11) $$\begin{array}{cccccc} \sum_{r\in R}\eta_{r,v,\tau } & \leq 1 & \; & \forall v\in V,\forall \tau & \; & (\mathrm{node}\;\mathrm{occupancy}:\;\mathrm{one}\;\mathrm{robot}\;\mathrm{per}\;\mathrm{node}\;\mathrm{per}\;\mathrm{time}\;\mathrm{step}) \end{array}$$

(12) $$\begin{array}{cccccc} \eta_{r,i,\tau }+\eta_{r^{\prime },j,\tau +1}+\eta_{r,j,\tau +1}+\eta_{r^{\prime },i,\tau } & \leq 3 & \; & \forall \left(i,j\right)\in E,\;r\neq r^{\prime },\forall \tau & \; & (\mathrm{edge}/\mathrm{swap}:\;\mathrm{no}\;\mathrm{head}\text{-}\mathrm{on}\;\mathrm{traversal}) \end{array}$$

where each robot’s route begins at $p_{r}^{0}$ and ends at $p_{r}^{\mathrm{end}}$, and the per-robot energy terms are given by the models in Section 3.4. The binary space-time occupancy variable $\eta_{r,v,\tau }=1$ indicates that robot *r* occupies node *v* at discrete time step τ; constraint (11) forbids two robots from occupying the same node at the same time (vertex collision), and constraint (12) forbids two robots from swapping positions across a shared edge in one step (edge/head-on collision), following the standard vertex- and edge-conflict conditions used in multi-agent path finding. In the present study, constraint (10) is evaluated as an outcome (reported energy) rather than enforced as a hard planning constraint, and the space-time occupancy constraints (11) and (12) are stated as part of the formal model but are enforced in the current implementation only through per-robot sequential planning with node reservation, rather than through a full joint conflict-resolution search (see Section 3.6); a complete treatment via conflict-based search is identified as future work. The assumptions below make this and the other simplifications explicit.

This formulation rests on the following assumptions, which bound the scope of the results in Section 4 and Section 5:

- Homogeneous robots: All robots share the same locomotion, computation, and communication characteristics (Section 3.4); heterogeneous platforms are not modeled in the present study.
- Single-hop offloading: A workload can be offloaded only to a robot within direct communication range $r_{c}$ (constraint (9)); multi-hop relaying is not modeled.
- Known task locations, dynamic execution decisions: The set of task locations for a scheduling iteration is known in advance, but the local-versus-offload decision $z_{j}$ and the resulting path are computed dynamically as the search proceeds, and are revised at the next scheduling iteration.
- Energy is tracked, not enforced: Constraint (10) is accounted for and reported as an outcome metric, but is not enforced during planning; robots do not fail or return to recharge when energy is exhausted.
- Static, known environment: The operational area and obstacle layout are assumed static and known; dynamic obstacles and localization uncertainty are not modeled.

The augmented A* algorithm of Section 3.5 is a heuristic solver for this model: it constructs each robot’s route (the *y* variables) while deciding $z_{j}$ inline at each node from the cheaper of local and offload cost, rather than solving (5)–(10) to global optimality (see Section 3.6 for what is and is not guaranteed).

### 3.3. Environment and Task Model

Let G=(V,E) be an undirected graph where each vertex v∈V represents a task location (waypoint) and each edge (i,j)∈E has an associated travel cost $d_{ij}$ (e.g., Euclidean distance or expected traversal time). Each robot in the swarm must visit a subset of *V* to perform computational tasks, where each task at *v* incurs a workload $w_{v}$ (e.g., data processing time).

### 3.4. Composite Cost Formulation

To trade off travel, computation, and communication, we define for each node *v* the costs:

(13) $$\begin{array}{cc} C_{travel}\left(i,j\right) & =\alpha \;d_{ij} \end{array}$$

(14) $$\begin{array}{cc} C_{local}\left(v\right) & =\beta \;w_{v} \end{array}$$

(15) $$\begin{array}{cc} C_{offload}\left(v\right) & =\gamma \min_{v^{\prime }\in N\left(v\right)}c_{vv^{\prime }} \end{array}$$

where α,β,γ>0 are weighting factors, N(v) is the set of neighboring robots or base stations reachable from *v*, and $c_{vv^{\prime }}$ is the communication cost (in energy or time) of offloading workload $w_{v}$ from *v* to neighbor $v^{\prime }$. At run time, the effective compute cost at *v* is

(16) $$C_{compute}\left(v\right)=\min\left\{C_{local}\left(v\right),\;C_{offload}\left(v\right)\right\}$$

Thus, each path segment incurs $C_{travel}$ plus the chosen compute cost $C_{compute}$. Evaluated from the perspective of a specific robot–task pair, this same per-decision cost is written

(17) $$C_{total}\left(r_{i},j\right)=\alpha \;d_{i,j}+\beta \;c_{j}+\gamma \;\lambda_{i,j},$$

where $d_{i,j}$ is the distance between robot $r_{i}$ and task *j*, $c_{j}$ is the local computation cost at node *j*, and $\lambda_{i,j}$ is the estimated communication cost to offload from $r_{i}$ to node *j*; $\lambda_{i,j}$ plays the same role as $c_{vv^{\prime }}$ above, instantiated by the communication model below for the specific pair $\left(r_{i},j\right)$.

#### 3.4.1. Communication Cost

Robots communicate over Bluetooth Low Energy (BLE) links. Offloading a workload with input data size $D_{v}$ (bits) from node *v* to neighbor $v^{\prime }$ requires transmission time $t_{vv^{\prime }}=D_{v}/R_{vv^{\prime }}$, where $R_{vv^{\prime }}$ is the effective BLE data rate of the link. The associated communication energy is

(18) $$c_{vv^{\prime }}=P_{tx}\;t_{vv^{\prime }}+E_{conn},$$

where $P_{tx}$ is the radio transmit power and $E_{conn}$ is a fixed per-transfer connection overhead. Links are considered available only within the BLE communication range $r_{c}$; for $∥p_{v}-p_{v^{\prime }}∥>r_{c}$, $c_{vv^{\prime }}=\infty$. The simulator uses a constant (distance-independent) link rate $R_{vv^{\prime }}=R=1\;\mathrm{Mbps}$ for all robot pairs within range; consequently only link availability, not throughput, depends on distance. The remaining parameters are $P_{tx}=0\;\mathrm{dBm}\approx 1\;\mathrm{mW}$, $E_{conn}=0.5\;\mathrm{mJ}$, and $r_{c}=10\;m$.

#### 3.4.2. Computation Energy

Executing workload $w_{v}$ locally consumes $E_{cpu}\left(v\right)=\kappa \;w_{v}$, where κ is the platform-specific energy per unit workload. The value and units of κ used in the simulator was 0.8 J per second of processing per cycle at our simulated CPU frequency.

#### 3.4.3. Locomotion Energy

Traversing an edge (i,j) consumes $E_{move}\left(i,j\right)=e_{m}\;d_{ij}$, where $e_{m}$ is the locomotion energy per meter, which in our case was 0.15 J/m.

#### 3.4.4. Total Energy

The total energy consumption reported in Section 4 is the sum over all robots of locomotion, local computation, and communication energy:

(19) $$E_{total}=\sum_{r}\left(\sum_{\left(i,j\right)\in \pi_{r}}E_{move}\left(i,j\right)+\sum_{v\in L_{r}}E_{cpu}\left(v\right)+\sum_{\left(v,v^{\prime }\right)\in O_{r}}c_{vv^{\prime }}\right),$$

where $\pi_{r}$ is the path traversed by robot *r*, $L_{r}$ the set of tasks it executed locally, and $O_{r}$ the set of offloads it performed.

#### 3.4.5. Weighting Factors: Interpretation, Units, and Normalization

The three terms of the composite cost are physically heterogeneous: $d_{ij}$ is a distance (m), $w_{v}$ (equivalently $c_{j}$) is a processing workload (s of compute), and $\lambda_{ij}$ (equivalently $c_{vv^{\prime }}$) is a communication cost (J, via Equation (18)). We distinguish two possible interpretations of α,β,γ, and state plainly which one applies to this study. In a *calibrated* design, each weight would carry the reciprocal unit of its term (α in cost per meter, β in cost per second, γ in cost per joule), chosen so that all three contributions map to a common physical scale such as joules; under that interpretation the weighted sum would itself be a physical quantity. That is *not* what was done here. In this study, α=1, β=2, γ=3 are bare numeric multipliers applied to the raw, unnormalized numeric values of the three terms; no per-component normalization (e.g., min–max or z-score) was applied, and the values were fixed manually during development, without a calibration procedure, to bias the search away from communication and offloading overhead relative to travel. As implemented, the weights therefore do not perform unit conversion, and $C_{total}$ (Equation (17)) is a unitless heuristic ranking score: it is meaningful for comparing candidate decisions within the search, but its magnitude has no physical interpretation. The total-energy results reported in Section 4 are computed independently, from Equations (18) and (19) directly, and are not derived from α,β,γ. Because the terms are unnormalized, the numeric ratio 1:2:3 does not by itself imply that communication is weighted three times as heavily as travel in effect; the effective influence of each term also depends on its raw numeric range in the simulator. We regard the absence of normalization and the manual, uncalibrated selection of α,β,γ as limitations of the present study, and identify the calibrated interpretation above, via per-component normalization plus grid-search or learned weight selection, as the concrete target of future work (Section 5).

#### 3.4.6. Swarm-Level Aggregate

For analyzing spatial effects at the level of a full scheduling iteration rather than a single decision (Section 4), the per-decision costs above are aggregated as

(20) $$\Phi_{\mathrm{sched}}=\sum_{i=1}^{N}\left(\rho_{i}T_{i}+\delta_{i}D_{i}\right),$$

where $T_{i}$ is the number of tasks assigned to robot *i*, $D_{i}$ is its cumulative travel distance, $\rho_{i}$ is its computational cost per task (accumulating the $\beta w_{v}$ or offload terms of its assigned tasks), and $\delta_{i}$ is a weighting factor accumulating its travel terms. Equations (17) and (20) therefore describe the same cost structure at two levels of aggregation: per robot–task decision, and per scheduling iteration.

### 3.5. Augmented A* Algorithm

The presented algorithm augments classical A* path planning with an in-line decision on where to execute per-node computation (locally or by offloading). Each transition cost combines three nonnegative components: a travel cost scaled by α ($C_{\mathrm{travel}}=\alpha d_{uv}$), a local compute cost scaled by β ($C_{\mathrm{local}}=\beta w_{v}$), and an offload cost $\gamma \min_{v^{\prime }\in N\left(v\right)}c_{vv^{\prime }}$, which is set to +∞ when no neighbor is available. For the tentative cost-to-come, the algorithm takes whichever of the local and offload costs is cheaper, and it records that choice for the node whenever the node’s *g*-score improves.

Algorithm 1 extends classical A* by embedding this per-node compute–communication trade-off directly into the path expansion step. At each expansion, the algorithm evaluates a composite incremental cost

(21) $$C\left(u,v\right)=\alpha d_{uv}+\min\left\{\beta w_{v},\;\gamma \min_{v^{\prime }\in N\left(v\right)}c_{vv^{\prime }}\right\}$$

which captures travel effort, local processing cost, and the best-case offloading cost, respectively, allowing the planner to choose opportunistically between local computation and offloading, conditioned on the current neighborhood connectivity and workload at node *v*.

**Figure 3:**
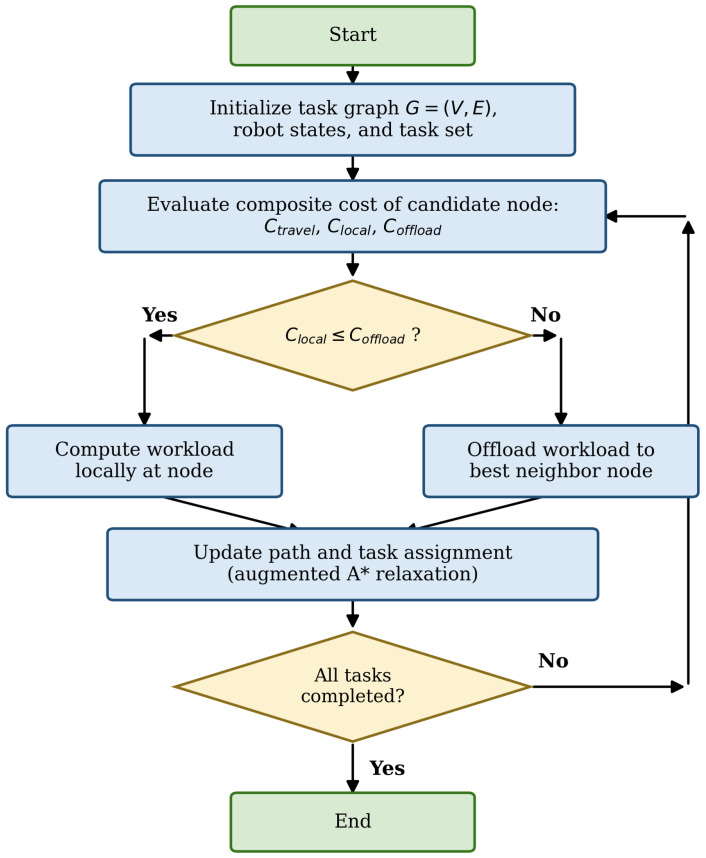
Workflow of the proposed methodology. At each candidate node the composite cost components are evaluated; the local-versus-offload decision is taken once per node (Yes: execute locally; No: offload to the best reachable neighbor), after which the path and task assignment are updated and the loop repeats until all tasks are completed.

Figure 3 summarizes this decision loop. During relaxation, the decision map is updated greedily, recording the lower-cost execution modality (local or offload) whenever a node’s *g*-score improves; this makes compute decisions path-dependent and locally optimal, a property analyzed further in Section 3.6. In rapidly varying communication environments, stale or noisy offloading cost estimates may necessitate re-planning, or the addition of uncertainty margins to γ to suppress oscillatory task reassignment.

**Figure 4:**
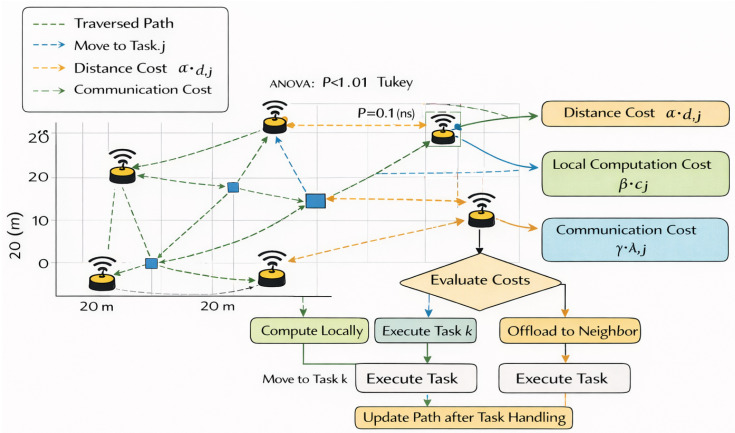
Per-robot view of the local-versus-offload decision: distance, computation, and communication costs are evaluated jointly before a robot commits to an execution mode.

An equivalent, per-robot view of the same decision logic, in which distance, local-computation, and communication costs are each evaluated before a robot commits to local execution or neighbor offloading, is shown in Figure 4.

Algorithm 1 is the inner, single-query routine: it plans one robot’s path between a start and a goal while deciding execution placement along the way. The complete multi-robot task planning process, which the experiments in Section 4 evaluate, wraps this routine in an outer allocation loop, formalized as Algorithm 2. Per scheduling iteration, the loop (i) ranks all robot–task candidate pairs by the composite cost of Equation (17), using current positions and neighborhoods; (ii) commits assignments greedily from the top of the ranking, respecting each robot’s capacity $Q_{r}$ and the single-assignment constraint (6); (iii) plans routes sequentially in the resulting priority order via Algorithm 1, with each robot treating the space-time cells reserved by higher-priority robots as blocked (constraints (11) and (12)); and (iv) after execution of the current assignments, re-enters the loop with updated positions, queues, and neighborhoods, so that placement decisions are revised as topology changes rather than fixed at dispatch time (the closed loop of Section 3.1).

We flag precisely where centralization enters this process, since it is easy to conflate with the routing step. Step (i), constructing C over *all* pairs (r,j)∈R×T and sorting it, requires visibility of every robot’s state and every outstanding task simultaneously; as written, this is a centralized computation, performed by a coordinator (or reconstructed identically by every robot from a full swarm-wide broadcast), not a decentralized one. This is distinct from step (iii), where each robot’s own route and compute-placement decision (Algorithm 1) uses only the neighborhood information described in Section 3.7. The architecture evaluated in this paper is therefore centralized assignment, decentralized execution: task-to-robot matching is computed with global information, while the resulting per-robot routing and local-versus-offload decisions are computed locally.

### 3.6. Computational Complexity

The heuristic $h\left(u\right)=\alpha \;{\mathrm{dist}}_{euclid}\left(u,g\right)$ uses only the travel term, so provided $\alpha d_{uv}$ lower-bounds the true travel cost, *h* is admissible and consistent with respect to the travel component. It is important to be precise about what this does and does not guarantee. Because the compute and communication terms enter only through edge relaxation and not through *h*, the search is not misled into pruning optimal-travel paths, and the algorithm retains the same asymptotic time complexity as standard A* (i.e., O(|E|log|V|) with a binary heap), with a modest constant-factor overhead for evaluating $C_{l}$ and $C_{o}$ at each neighbor. We do not claim global optimality of the joint routing-and-placement problem. Two effects break such a claim: (i) the compute-placement decision at each node is made greedily from the locally cheaper of local execution and offloading, without lookahead over how that choice affects downstream costs; and (ii) the offload cost $c_{vv^{\prime }}$ depends on which neighbors are in range along the chosen path, so the communication state is itself path-dependent, and the edge weights are therefore not fixed a priori as a classical shortest-path proof would require. The algorithm should thus be understood as a well-motivated heuristic that is admissible in its travel component and locally optimal in its placement decisions, not as an exact solver for the joint problem.

Consistent with the analysis above, the algorithm returns a path that is optimal with respect to the composite edge-cost objective it searches over, but the compute-placement decisions embedded in that objective are locally greedy, so the returned solution is not guaranteed optimal for the joint routing-and-placement problem. From an energy efficiency standpoint, the algorithm favors regions of the graph with favorable communication neighborhoods, since low offloading costs reduce composite edge weights and thus *f*-scores; this behavior presupposes that communication costs $c_{vv^{\prime }}$ are either precomputed or accurately estimated, and inaccuracies in these estimates can degrade the coherence of the joint path and compute-mode optimization. Whether these theoretical properties translate into measured energy and time savings is evaluated empirically in Section 4.

Collision avoidance is handled in the current implementation through sequential per-robot planning with node reservation: robots are planned in a fixed priority order, and each robot treats the reserved space-time cells of already-planned robots (constraints (11) and (12)) as blocked, which guarantees vertex- and edge-conflict-free plans at the cost of prioritized (rather than jointly optimal) coordination. A full joint treatment via conflict-based search, which resolves conflicts by branching rather than fixing a priority order, would improve coordination quality and is a natural next step. Other extensions, not evaluated in this paper, include online learning of α,β,γ from feedback and clustered offloading that batches multiple workloads to a single neighbor. The modular cost structure would also allow the $C_{o}$ term to incorporate queueing latency, stochastic link reliability, or multi-hop offloading penalties without changing the search procedure itself.

The complexity above describes the per-robot routing routine (Algorithm 1). The outer assignment loop (Algorithm 2) has its own cost, dominated by constructing and sorting the candidate set C of all robot–task pairs: with N=|R| robots and M=|T| outstanding tasks, |C|=NM, so ranking costs O(NMlog(NM)) per scheduling iteration, in addition to the *N* subsequent calls to Algorithm 1. As discussed in Section 3.7, this ranking step is centralized; a distributed auction-based alternative would trade this global sort for multiple rounds of local message passing, at a communication cost not analyzed.

### 3.7. Decentralization and Information Exchange

The framework is decentralized in its execution phase, but not, as currently implemented, in its assignment phase; we state this split precisely rather than describe the whole framework as decentralized. Once a robot has been assigned a task by Algorithm 2, its routing and local-versus-offload decision (Algorithm 1) uses only information obtainable from its local neighborhood, rather than from a central controller with global state. Concretely, the only non-local quantities this local routine requires are (i) the identities and positions of in-range neighbors N(v) and (ii) their advertised availability/queue state, both of which are exchanged over the same BLE links used for offloading (Section 3.4). We assume periodic neighbor discovery: within each scheduling iteration, a robot broadcasts a lightweight beacon (its position and current queue length) and listens for beacons from robots within $r_{c}$, so that N(v) and the offload cost $c_{vv^{\prime }}$ can be estimated locally. No global map of the swarm is assembled for this step, and no robot requires connectivity to robots outside its communication radius. Sequential priority-based conflict resolution (Section 3.6) requires robots to exchange their reserved space-time cells with higher-priority neighbors, which is likewise a neighborhood-local exchange.

The assignment phase is different. Algorithm 2 ranks every robot-task pair by composite cost before committing assignments (Section 3.5), which requires simultaneous visibility of all robots’ states and all outstanding tasks; this is a centralized computation as implemented, performed either by a coordinator or reconstructed identically by every robot from a full swarm-wide broadcast, and it does not use only neighborhood-local information. We flag this as the precise scope boundary of the “decentralized” claim: decentralization in this paper describes how a robot executes an assignment it has already received, not how that assignment was computed.

Three caveats bound the resulting claims. First, task assignment (Algorithm 2, Step 2–3) requires global visibility as described above; a genuinely decentralized alternative, for example a distributed auction along the lines of CBBA [11,12], in which robots bid on tasks using only locally estimated costs and converge to a conflict-free assignment through local message exchange, has not been implemented or evaluated here, and is identified as a concrete direction for future work (Section 5). Second, the priority ordering used for conflict resolution during routing is a global convention (e.g., by robot index) rather than something negotiated purely locally; a fully local negotiation protocol is likewise left to future work. Third, the beaconing overhead and its effect on the communication-energy budget are modeled only through the per-transfer cost $E_{conn}$ (Equation (18)) and are not separately profiled. We report the per-query computational complexity of the local routing step in Section 3.6, but the end-to-end decision latency, including neighbor-discovery round-trips on physical BLE hardware and the coordination overhead of the centralized assignment step, has not been measured; accordingly, we do not attach a wall-clock real-time guarantee to the method (Section 5.2).

### 3.8. Experimental Setup

The augmented A* search of Section 3.5 is evaluated in two complementary experiments, both reported in Section 4: (i) an assessment of how the spatial distribution of robots affects the aggregate scheduling cost $\Phi_{\mathrm{sched}}$ (Equation (20)), and (ii) a comparison of the full framework against two decoupled baselines that each ignore one component of the composite cost.

**Figure 5:**
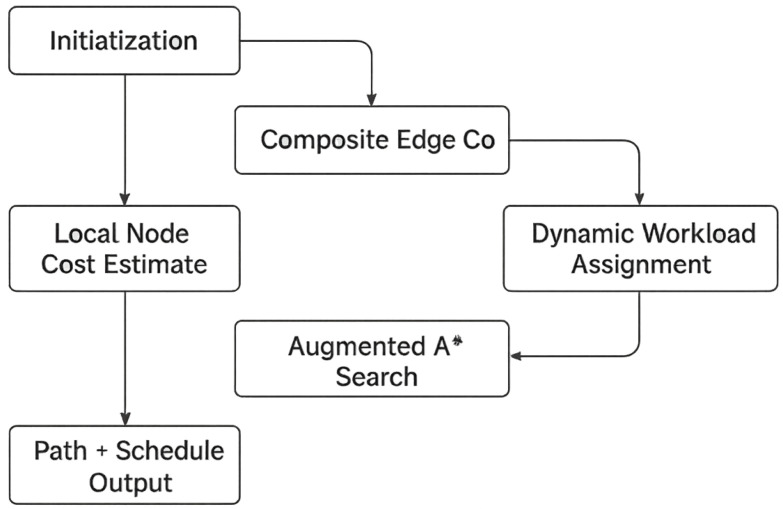
Closed-loop structure linking composite cost evaluation, dynamic workload assignment, and the augmented A* search, used throughout both experiments.

**Figure 6:**
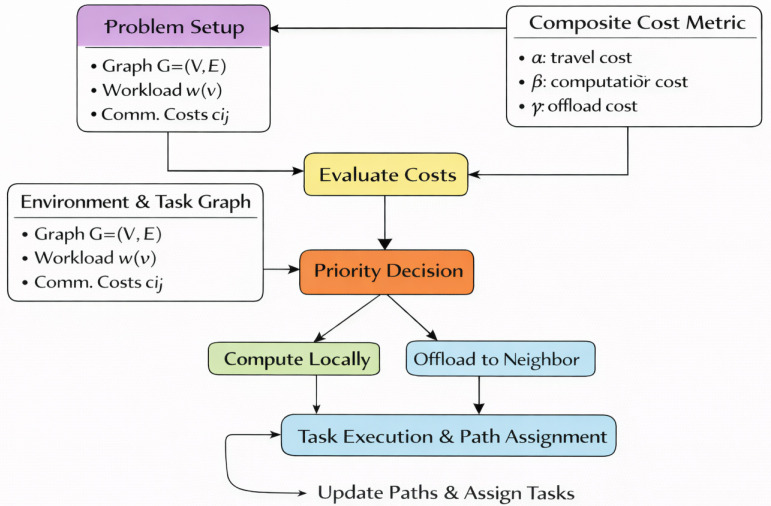
Priority-based view of the same pipeline: candidate robot–task assignments are ranked by composite cost before path and compute-mode assignment.

Figure 5 and Figure 6 illustrate this shared experimental pipeline from two complementary angles: the former as an iterated closed loop between cost evaluation and search, the latter as a priority ranking over candidate robot–task assignments prior to path and compute-mode assignment. Both experiments use the same underlying algorithm (Section 3.5) and cost model (Section 3.4); they differ only in what is varied (spatial layout versus planning strategy) and which metrics are reported.

#### 3.8.1. Spatial-Distribution Experiment

A swarm of N=30 robots is deployed within a 20×20 m operational area under three deployment strategies: clustered, uniform, and random. Computational tasks are assigned to the nearest robot with sufficient processing capacity, and the communication-aware heuristic jointly determines robot trajectories and computation-placement decisions while minimizing $\Phi_{\mathrm{sched}}$.

#### 3.8.2. Baseline-Comparison Experiment

We deploy N=25 IoT robots in a 30×30 m environment with 10 task locations, randomly placed at the start of each run. Each location generates task instances at a fixed rate of one new task every 2 s, with all 10 locations generating instances at the same rate, for a total of 200 task instances per run (20 instances per location); no instances are present before t=0. Each robot has a local task queue and a BLE communication module governed by the models of Section 3.4. The simulation platform was a custom Python 3.14.6 simulator with a fixed time step of 0.1 s, a fixed robot speed of 0.5 m/s, a uniform task workload across tasks, and 20 fixed random seeds (0–19) across trials for reproducibility. Three strategies are compared:

1. Baseline 1 (Path only): Each robot repeatedly selects the nearest unassigned task by travel cost $\alpha d_{ij}$ and executes every task locally; communication and workload terms are excluded from the decision.
2. Baseline 2 (Workload only): Tasks are assigned to balance per-robot queue lengths, without considering travel distance or communication cost; execution is local.
3. Proposed Heuristic: Joint co-optimization of path, workload, and communication costs as described in Section 3.5.

Each experiment is repeated 20 times with randomized initial robot positions.

#### 3.8.3. Performance Metrics

Both experiments report the following:

- Total Energy Consumption (J): The cumulative energy expended by the swarm for both locomotion and communication (Equation (19)).
- Average Task Completion Time (s): The elapsed time from task assignment to completion, averaged across all tasks.
- Load Imbalance Factor (LIF): The standard deviation, across the swarm, of the number of task instances assigned per robot over the course of a run. With 200 task instances (10 locations × 20 instances each, Section 3.8) distributed over 25 robots, the theoretical maximum standard deviation of tasks-per-robot is ≈39.2 (one robot receiving all 200, the rest none), so the reported values (1.9–6.7) are well within the feasible range once the full 200-instance count, rather than the 10 task locations, is used as the basis for LIF.

## 4. Results

This section reports the outcomes of the two experiments described in Section 3.8: the effect of spatial distribution on scheduling cost (Section 4.1), and the comparison of the proposed heuristic against the two decoupled baselines (Section 4.2), including an analysis of how the composite cost decomposes across its three components (Section 4.3).

### 4.1. Effect of Spatial Distribution on Computational Cost

**Figure 7:**
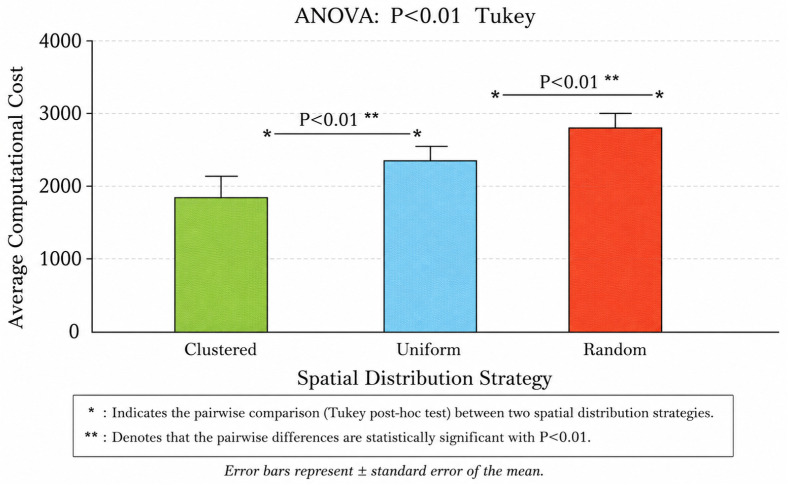
Average computational cost for clustered, uniform, and random spatial deployment strategies. Error bars represent the corresponding confidence intervals.

Figure 7 summarizes the average computational cost, $\Phi_{\mathrm{sched}}$ (Equation (20)), obtained for the three spatial deployment strategies defined in Section 3.8.

The clustered deployment consistently produces the lowest computational cost. Because robots remain geographically close to task-dense regions, communication distances are shorter, computational workloads are exchanged more efficiently, and fewer long-range task reallocations are required, reducing both communication overhead and overall scheduling cost.

The uniform deployment exhibits intermediate performance. Although robots are evenly distributed throughout the workspace, the regular spacing does not always coincide with the spatial distribution of computational demand; robots consequently perform a greater number of workload transfers than in the clustered configuration, producing a moderate increase in cost.

The random deployment yields the highest computational cost. Irregular robot spacing creates heterogeneous communication links and uneven workload distribution, increasing both travel distance and communication overhead, so that computation-placement decisions become less efficient and additional scheduling iterations are often required to balance the workload.

Each deployment strategy was evaluated over several independent runs. A one-way ANOVA indicates statistically significant differences among the three deployment strategies. Tukey’s post-hoc analysis confirms that all pairwise comparisons are statistically significant, with the largest difference observed between the clustered and random deployments.

**Figure 8:**
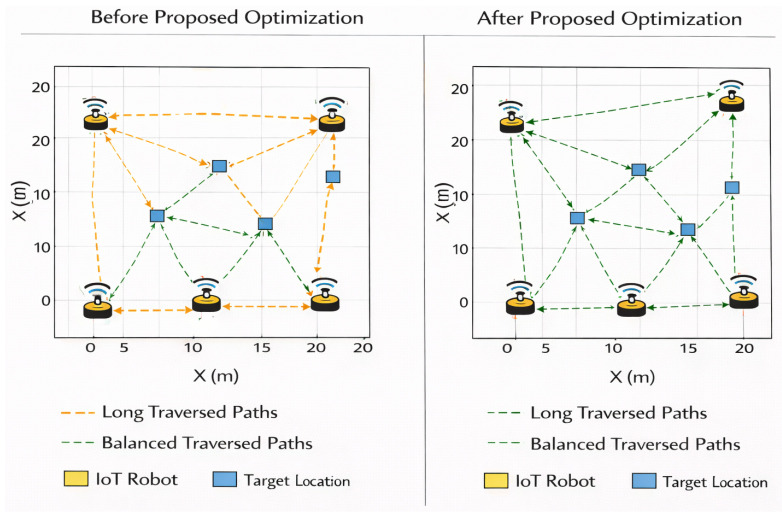
Robot trajectories before and after communication-aware co-optimization.

The influence of the proposed communication-aware heuristic on robot trajectories, for the same spatial-distribution experiment, is shown in Figure 8.

Before optimization, robots follow relatively long and frequently overlapping trajectories, leading to unnecessary travel, increased communication distance, and uneven computational load distribution; such trajectories require repeated long-range communication and increase the overall energy required to complete the mission. After applying the proposed framework, robot trajectories become more localized and coordinated: the planner preferentially assigns nearby tasks while selecting the more energy-efficient execution mode according to the composite cost model, so that workload distribution becomes more balanced, long-distance communication is reduced, and unnecessary robot movement is avoided. The resulting trajectory change simultaneously decreases travel distance, communication overhead, and computational imbalance across the swarm, evidencing the benefit of integrating navigation planning with communication-aware computation placement rather than optimizing each component independently.

### 4.2. Comparison with Baseline Strategies

**Figure 9:**
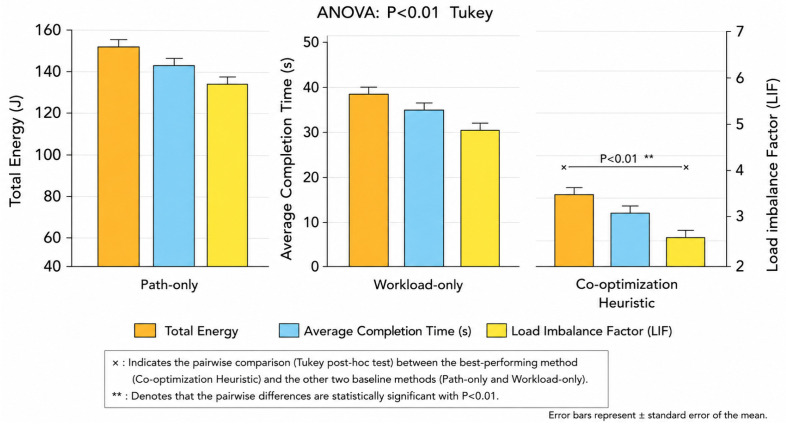
Performance of the co-optimization heuristic and baseline strategies.

Figure 9 compares the proposed co-optimization heuristic against both baselines defined in Section 3.8, across total energy consumption, average task completion time, and load imbalance factor (LIF).

The path-only strategy achieves modest reductions in travel-related energy but records the highest overall energy consumption and load imbalance, as computational demand remains unevenly distributed across the swarm. The workload-only strategy improves load balancing but disregards spatial efficiency, resulting in excess traversal overhead and longer completion times. The proposed co-optimization heuristic outperforms both baselines across all three metrics, achieving the lowest total energy consumption, the shortest average completion time, and the smallest load imbalance factor. With n=20 replications per strategy, one-way ANOVA confirms significant between-strategy differences for each metric, and Tukey’s post-hoc test confirms that all pairwise improvements of the proposed heuristic over both baselines are statistically significant. Table 2 reports the mean values over the 20 trials.

Communication-aware path planning reduces total energy use by approximately 22% relative to Baseline 1 and 9% relative to Baseline 2. Average completion time is roughly 20% shorter than the path-only baseline. The load imbalance factor is substantially lower than both baselines, indicating a fairer distribution of tasks while maintaining low communication and travel costs. These results indicate that decoupling path planning from workload assignment forces a trade-off between energy efficiency and execution latency that the jointly optimized heuristic avoids, by handling spatial routing and computational distribution within a single search procedure.

**Figure 10:**
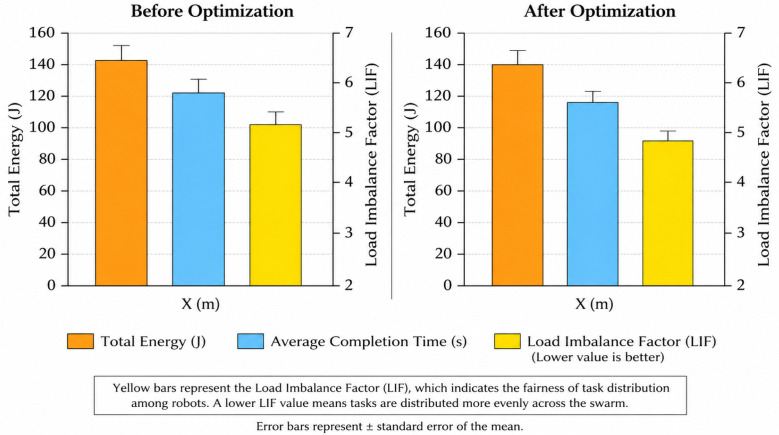
System performance before and after applying the co-optimization heuristic.

Figure 10 presents the same comparison as a direct before/after view of system performance when the proposed framework is applied to the baseline-comparison experiment.

Prior to optimization, the swarm exhibits higher energy consumption and longer task completion times, attributable to poor task sequencing and uneven distribution of computational load; the elevated LIF reflects persistent congestion at overloaded robots, which creates localized bottlenecks and increases reliance on long-range communication. After optimization, all three indicators improve consistently: total energy consumption decreases as robots prioritize co-located task execution and short-range communication, average completion time shortens due to fewer replanning events and smoother task-to-task transitions, and the marked reduction in LIF confirms a more equitable workload distribution across the swarm.

### 4.3. Decomposition of the Composite Cost

**Figure 11:**
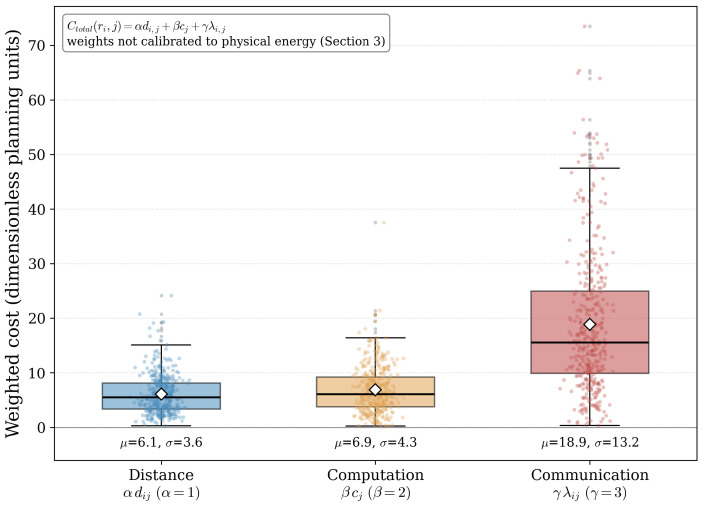
Distribution of the three weighted cost components (distance term $\alpha d_{ij}$, computation term $\beta c_{j}$, communication term $\gamma \lambda_{ij}$; α=1, β=2, γ=3) evaluated at each robot–task scheduling decision. Boxes show the interquartile range with the median (solid line) and mean (diamond); points are individual decisions. Values are dimensionless planning-cost units used internally by the search heuristic rather than physical energy in joules, since these weights are uncalibrated planning parameters (Section 3.4) and independent of the physical energy accounting in Table 2 and Equation (19).

Figure 11 decomposes the composite cost of Equation (17) into its three weighted components, distance ($\alpha \cdot d_{ij}$), computation ($\beta \cdot c_{j}$), and communication ($\gamma \cdot \lambda_{ij}$), as evaluated during the baseline-comparison experiment.

The communication term dominates the overall objective, exhibiting the highest mean value and variance across all evaluated configurations. The comparatively small distance contribution indicates that, in this experiment, purely geometric path optimization yields diminishing returns once basic spatial coherence is established. The elevated communication cost, by contrast, reflects the swarm’s sensitivity to link quality, offloading delay, and neighborhood connectivity. The computation term occupies an intermediate position, capturing the trade-off between local execution and offloading: processing tasks locally avoids communication overhead but imposes greater energy and time costs on heavily loaded nodes. These observations support the decision, motivated in Section 3.4, to weight communication explicitly in the composite metric; because these weights are uncalibrated (Section 3.4), the values in Figure 11 should be read as evidence for the qualitative ranking of cost sources, not as calibrated energy magnitudes.

## 5. Discussion

### 5.1. Interpretation of the Results

Taken together, the results in Section 4 support the central design claim of Section 3: that pricing routing and computation placement inside a single search step, rather than in separate stages, yields measurable gains over decoupled alternatives. The spatial-distribution experiment (Section 4.1) shows that this benefit is not uniform across deployments, clustered swarms benefit most because short communication distances make the offload term cheap, while random deployments benefit least because irregular connectivity raises the offload term relative to local execution. The baseline comparison (Section 4.2) isolates the same effect from a different angle: a path-only planner and a workload-only scheduler each optimize one term of the composite cost while ignoring the others, and both are outperformed by the joint heuristic on every metric, energy, time, and fairness, rather than trading one metric for another. The cost decomposition (Section 4.3) explains why: communication, not distance, is the dominant and most variable term in this environment, which is consistent with the observation that spatial clustering (which primarily reduces communication cost) produces a larger effect than path shortening alone would predict.

Accounting explicitly for communication cost, alongside path and computation factors, therefore appears to be the specific mechanism behind the observed improvements, rather than the joint optimization being beneficial for generic reasons. This matters particularly for low-power IoT robots, where avoiding expensive data transfers and managing computational load can substantially extend operational lifetime, though this extension itself was not directly measured (see Limitations in Section 5.2 below).

### 5.2. Limitations

We highlight the following limitations of the present study, several of which bound the claims made above and in Section 1.

- Uncalibrated cost weights: The weights α=1, β=2, γ=3 used throughout (Section 3.4) were set manually and not calibrated against the physical energy models or selected via a systematic procedure. The qualitative ranking of cost components in Section 4.3 is therefore more trustworthy than the specific magnitudes shown; a different, calibrated weighting could shift the relative size of the three terms, though the total-energy results in Table 2 are computed independently of these weights and are not subject to this limitation.
- Incomplete statistical reporting: The ANOVA results reported in Section 4.1 state significance but not exact *F*-statistics, degrees of freedom, or per-strategy standard deviations; Table 2 similarly reports mean values without dispersion.
- Centralized assignment, decentralized execution: As detailed in Section 3.7, only the routing and local-versus-offload execution decisions (Algorithm 1) are computed from local neighborhood information; the task-assignment step (Algorithm 2) ranks all robot–task pairs using swarm-wide visibility and is therefore centralized as implemented. A fully decentralized assignment mechanism, e.g., a distributed auction such as CBBA, has not been implemented or evaluated.
- Single-hop communication and small swarms: The communication model (Section 3.4) permits only single-hop offloading within a 10 m range, and the experiments evaluate swarms of 25–30 robots in a single environment size (20–30 m per side). Larger swarms, longer ranges, or multi-hop relaying may change the relative cost of communication versus computation, and were not tested.
- Limited dynamism: The evaluation varies spatial deployment and compares against static baselines, but does not vary swarm size, task count, or initial battery level, and does not model dynamic obstacles, localization uncertainty, or time-varying communication topology (Section 3.2). The framework’s behavior under these forms of dynamism, which motivated its design, is therefore not yet directly demonstrated.
- Simulation only: All results are obtained in a custom Python simulator (Section 3.8). The rover prototype shown in Figure 1 was not used to generate any of the reported results, and no claim in this paper is based on physical hardware. Consequently, we do not claim demonstrated real-world suitability; whether the simulated communication and energy models transfer to physical BLE hardware, and whether the algorithm’s low asymptotic overhead (Section 3.6) translates into acceptable wall-clock latency on embedded compute, remain open questions.

### 5.3. Future Work

Motivated directly by the limitations above, future work will proceed in five directions. First, the manually selected weights α,β,γ will be replaced with a systematic calibration procedure, for example a grid search against the physical energy model or adaptive weighting via reinforcement learning. Second, the sensitivity of the reported gains to swarm size, task count, and initial battery level will be evaluated directly, extending the single-configuration comparisons of Section 4 to parameter sweeps. Third, dynamic replanning mechanisms will be incorporated to accommodate time-varying communication topology and online task arrivals, moving beyond the static, known-topology assumption of Section 3.2. Fourth, the currently centralized assignment step (Algorithm 2) will be replaced with a distributed auction mechanism in the style of CBBA [11,12], in which robots bid on tasks from locally estimated costs and converge to an assignment through local message exchange, removing the swarm-wide visibility requirement identified in Section 3.7. Finally, the framework will be validated on the physical rover platform of Figure 1 and on heterogeneous robotic platforms more broadly, to assess its robustness under realistic sensing, localization, and wireless communication conditions that the present simulation does not capture.

## 6. Conclusions

The central question of this paper was whether pricing routing and computation placement inside a single search step, rather than resolving them in separate stages, changes swarm-level outcomes. The evidence in Section 4 answers this affirmatively for the settings tested: the joint heuristic outperformed both a path-only and a workload-only baseline on energy, completion time, and workload fairness simultaneously, and the cost decomposition traced this advantage to its explicit treatment of communication cost, the dominant and most variable term in the environments studied, rather than to the joint formulation being beneficial in some unspecified general sense.

That answer is deliberately bounded. As detailed in Section 5.2, the reported gains rest on manually chosen cost weights rather than a calibrated procedure, on swarms of 25–30 robots with single-hop, short-range communication, and on a simulator that has not yet been checked against the physical rover platform used to motivate the work. None of these are grounds to discount the result that joint optimization outperformed decoupled alternatives in the tested configurations; they are grounds to treat that result as a demonstration of the mechanism, not yet as a validated deployment strategy. Closing that distance, through weight calibration, sensitivity analysis across swarm size and battery level, dynamic replanning, and hardware validation, is the explicit agenda for future work set out in Section 5, rather than an open-ended list of possible extensions.

More generally, the results suggest that swarm coordination frameworks which treat communication cost as a first-class, topology-sensitive term inside the planning search, rather than as a fixed distance proxy or a post-hoc constraint, are worth the added modeling effort in communication-constrained deployments, where the experiments here indicate the communication term is where most of the achievable savings reside.

## Figures and Tables

**Table 1:** Positioning against representative method families. ✓: addressed; ∼: partially/assumed given; ×: not addressed.

Method Family	Travel Routing	Compute Placement	Comm. Cost
MRTA/task allocation [29,32]	∼	×	×
CBBA/auction [11,12]	∼	×	∼
MEC offloading [33,35,36,37]	∼	✓	✓
Comm. aware routing/coding [34,38]	×	×	✓
Joint alloc. + trajectory [30]	✓	×	∼
This work	✓	✓	✓

**Table 2:** Comparison of the co-optimization heuristic with baseline strategies. Values are mean over n=20 randomized trials.

Strategy	Energy (J)	Time (s)	LIF
Baseline 1 (Path only)	152.4	38.2	6.7
Baseline 2 (Workload only)	131.0	34.5	3.2
Proposed Heuristic	118.6	30.8	1.9

## Data Availability

The simulation configurations, random seeds, initial robot positions, task locations, and analysis scripts used in this study are available from the corresponding author upon reasonable request. No real-world or confidential deployment data were used in this study.

## References

[1] Al-Fuqaha A., Guizani M., Mohammadi M., Aledhari M., Ayyash M. (2015). Internet of Things: A survey on enabling technologies, protocols, and applications. IEEE Commun. Surv. Tutor..

[2] Liu J., Li C., Luo Y. (2024). Efficient resource allocation for IoT applications in mobile edge computing via dynamic request scheduling optimization. Expert Syst. Appl..

[3] Amin A.Z.M., Sahrani S. (2025). A Deep Reinforcement Learning Approach for Spectrum Management in Tri-Band Wi-Fi Networks. 2025 IEEE 7th Symposium on Computers & Informatics (ISCI).

[4] Xiong Y., Liu F., Cui Y., Yuan W., Han T.X., Caire G. (2023). On the fundamental tradeoff of integrated sensing and communications under Gaussian channels. IEEE Trans. Inf. Theory.

[5] Zhang T., Wang S., Li G., Liu F., Zhu G., Wang R. (2022). Accelerating edge intelligence via integrated sensing and communication. ICC 2022—IEEE International Conference on Communications.

[6] Nakabi T., Toivanen P. (2021). Deep reinforcement learning for energy management in a microgrid with flexible demand. Sustain. Energy Grids Netw..

[7] Dev K., Maddikunta P.K.R., Gadekallu T.R., Bhattacharya S., Hegde P., Singh S. (2022). Energy optimization for green communication in IoT using Harris Hawk’s optimization. IEEE Trans. Green Commun. Netw..

[8] Brambilla M., Ferrante E., Birattari M., Dorigo M. (2013). Swarm robotics: A review from the swarm engineering perspective. Swarm Intell..

[9] Mao Y., You C., Zhang J., Huang K., Letaief K.B. (2017). A survey on mobile edge computing: The communication perspective. IEEE Commun. Surv. Tutor..

[10] Sathish, Kumar L., Ahmad S., Routray S., Prabu A.V., Alharbi A., Alouffi B., Rajasoundaran S. (2022). Modern energy optimization approach for efficient data communication in IoT-based wireless sensor networks. Wirel. Commun. Mob. Comput..

[11] Dorigo M., Theraulaz G., Trianni V. (2021). Swarm robotics: Past, present, and future. Proc. IEEE.

[12] Liu Y., Passino K.M. (2004). Stable social foraging swarms in a noisy environment. IEEE Trans. Autom. Control..

[13] Nedjah N., Ribeiro L.M., Mourelle L.M. (2021). Communication optimization for efficient dynamic task allocation in swarm robotics. Appl. Soft Comput..

[14] Cui Y., Liu F., Jing X., Mu J. (2021). Integrating sensing and communications for ubiquitous IoT: Applications, trends, and challenges. IEEE Netw..

[15] Li X., Gong Y., Huang K., Niu Z. (2023). Over-the-air integrated sensing, communication, and computation in IoT networks. IEEE Wirel. Commun..

[16] Abdul-Qawy A., Alduais N.M., Saad A.M., Taher M.A., Nasser A.B., Saleh S., Khatri N. (2023). An enhanced energy efficient protocol for large-scale IoT-based heterogeneous WSNs. Sci. Afr..

[17] Peng G., Wang S., Huang Y., Huang T., Liu Y. (2022). Enabling deterministic tasks with multi-access edge computing in 5G networks. IEEE Commun. Mag..

[18] Cao X., Wang F., Xu J., Zhang R., Cui S. (2019). Joint computation and communication cooperation for energy-efficient mobile edge computing. IEEE Internet Things J..

[19] Chen L., Zhou S., Xu J. (2018). Computation peer offloading for energy-constrained mobile edge computing in small-cell networks. IEEE/ACM Trans. Netw..

[20] Shao J., Zhang J. (2020). Communication-computation trade-off in resource-constrained edge inference. IEEE Commun. Mag..

[21] Espinosa E., Gandara O., Lepe I., Guerrero A. (2018). Power Consumption Analysis of Bluetooth Low Energy Commercial Products and Their Implications for IoT Applications. Electronics.

[22] Yang K., Shi Y., Yu W., Ding Z. (2020). Energy-efficient processing and robust wireless cooperative transmission for edge inference. IEEE Internet Things J..

[23] Yan J., Bi S., Zhang Y.-J.A. (2022). Optimal model placement and online model splitting for device-edge co-inference. IEEE Trans. Wirel. Commun..

[24] Xu J., Chen L., Zhou P. (2018). Joint service caching and task offloading for mobile edge computing in dense networks. IEEE INFOCOM 2018—IEEE Conference on Computer Communications.

[25] Kreković D., Krivić P., Žarko I.P., Kušek M., Le-Phuoc D. (2025). Reducing communication overhead in the IoT-edge-cloud continuum: A survey on protocols and data reduction strategies. Internet Things.

[26] Bormann C., Castellani A.P., Shelby Z. (2012). CoAP: An application protocol for billions of tiny Internet nodes. IEEE Internet Comput..

[27] Chiang M., Zhang T. (2016). Fog and IoT: An overview of research opportunities. IEEE Internet Things J..

[28] Mokhtari M., Mohamadi P.H.A., Aernouts M., Singh R.K., Vanderborght B., Weyn M., Famaey J. (2025). Energy-aware multi-robot task scheduling using meta-heuristic optimization methods for ambiently-powered robot swarms. Robot. Auton. Syst..

[29] Ijaz A., Haghbayan H., Malik A., Nigussie E., Plosila J., Shakshuki E. (2025). Towards Optimizing Communication Cost in Energy Efficient IoT Devices for Swarm Robotics. Procedia Comput. Sci..

[30] Alqudsi Y., Makaraci M. (2025). Towards optimal guidance of autonomous swarm drones in dynamic constrained environments. Expert Syst..

[31] Raza U., Kulkarni P., Sooriyabandara M. (2017). Low Power Wide Area Networks: An overview. IEEE Commun. Surv. Tutor..

[32] Ferreira B.A., Petrović T., Orsag M., Dios J.R.M., Bogdan S. (2024). Distributed allocation and scheduling of tasks with cross-schedule dependencies for heterogeneous multi-robot teams. IEEE Access.

[33] Palieri M., Longo A., Edlund J., Vaquero T., Morrell B., Agha-mohammadi A., Guaragnella C. (2024). Swarm Manager: SDN-inspired computation offloading management in heterogeneous multi-robot networks. IEEE INFOCOM 2024—IEEE Conference on Computer Communications Workshops (INFOCOM WKSHPS).

[34] Yasser M., Shalash O., Ismail O. (2024). Optimized decentralized swarm communication algorithms for efficient task allocation and power consumption in swarm robotics. Robotics.

[35] Wu X., Dong J., Bao W., Zou B., Wang L., Wang H. (2024). Augmented intelligence of things for emergency vehicle secure trajectory prediction and task offloading. IEEE Internet Things J..

[36] Wu X., Wang H., Wei D., Shi M. (2020). ANFIS with natural language processing and gray relational analysis based cloud computing framework for real time energy efficient resource allocation. Comput. Commun..

[37] Wu X., Wang H., Tan W., Wei D., Shi M. (2020). Dynamic allocation strategy of VM resources with fuzzy transfer learning method. Peer-to-Peer Netw. Appl..

[38] Zhu R., Li W., Boukerche A., Yang Q. (2026). Energy-aware DRL-based dual-perception fountain codes for resource-constrained UASNs. IEEE Trans. Sustain. Comput..

